# A GPU‐based Monte Carlo model for water radiolysis under ultra‐high dose rate irradiation: Development and validation with MPEXS2.1‐DNA

**DOI:** 10.1002/mp.70071

**Published:** 2025-10-24

**Authors:** Shogo Okada, Koichi Murakami, Tamon Kusumoto, Katsuya Amako, Takashi Sasaki

**Affiliations:** ^1^ Computing Research Center High Energy Accelerator Research Organization (KEK) Tsukuba Ibaraki Japan; ^2^ Radiation Measurement Research Group National Institutes for Quantum Science and Technology (QST) Inage‐ku Chiba Japan

**Keywords:** CUDA, GPGPU, Monte Carlo simulation, ultra‐high dose rate irradiation, water radiolysis simulations

## Abstract

**Background:**

FLASH radiotherapy using ultra‐high dose rates (UHDR, > 40 Gy/s) demonstrates significant healthy‐tissue sparing while maintaining tumor‐control effectiveness. However, the underlying mechanisms remain unclear, with hypotheses suggesting that reductions in reactive oxygen species (ROS) yields could contribute to the FLASH effect. Direct experimental measurements of ROS dynamics under UHDR conditions are challenging, making Monte Carlo simulations valuable complementary tools for tracking individual water radiolysis species over time.

**Purpose:**

To enable a mechanistic investigation of ROS yield reductions under UHDR conditions, we developed a GPU‐based water radiolysis simulation platform capable of modeling key radiation chemistry processes.

**Methods:**

Using our MPEXS2.1‐DNA framework with step‐by‐step molecular tracking, we simulated sequential 55 MeV proton irradiation in a 1 × 1 × 1 µm^3^ target volume within a 2 × 2 × 2 µm^3^ water phantom filled with neutral pH water at dose rates ranging from 0.02 Gy/s to 500 Gy/s, with a total absorbed dose of 10 Gy. Simulations were performed under oxygenated (pO_2_ = 25%, 239.4 µM) and deoxygenated (pO_2_ = 0%) conditions without additional scavengers. Radiation chemical yields (G values, species/100 eV) of hydroxyl radicals, hydrated electrons, and hydrogen peroxide were calculated at time points from 1 ms to 1000 s post‐irradiation. Statistical analysis was performed using 1000 independent sequential proton irradiation scenarios per dose rate condition, processed in parallel on a single GPU. Results were compared with published experimental data.

**Results:**

The calculated dose rate dependence of the G values of hydroxyl radicals showed agreement with experimental data, with relative G values decreasing monotonically from 1.0 at 0.02 Gy/s to approximately 0.12 at 500 Gy/s at 10 ms post‐irradiation. Our simulations revealed that intertrack chemical reactions between neighboring proton tracks occurred, leading to decreases in the G values of hydroxyl radicals through enhanced radical‐radical interactions. The G values of hydrated electrons remained constant (G ≈ 2.5 species/100 eV) across all dose rates under deoxygenated conditions, consistent with experimental observations. Oxygen consumption followed a depletion rate of 0.028%/Gy (0.27 µM/Gy), in agreement with experimental measurements, but was insufficient to cause significant depletion at typical UHDR doses (10–40 Gy). Additionally, the calculated G values of hydrogen peroxide increased by 20% with dose rate (contrary to measured decreases of 20%–40%), suggesting the presence of competitive reaction pathways not included in current models.

**Conclusions:**

We developed a GPU‐based computational framework for water radiolysis simulations under UHDR conditions using MPEXS2.1‐DNA. Our step‐by‐step approach enabled spatially precise tracking of intertrack reactions among radiolysis species originating from different proton tracks, a capability not achievable with conventional simplified methods. Additionally, we demonstrated that large‐scale statistical analysis is computationally feasible under UHDR conditions through GPU acceleration. Our results successfully reproduced the experimental trends for the G values of hydroxyl radicals and hydrated electrons. Oxygen depletion rates also showed good agreement with experimental measurements. However, the discrepancy between simulated and measured G values of hydrogen peroxide indicates the need to incorporate additional competitive reactions, potentially including third‐order mechanisms, for future UHDR modeling studies.

## 1 INTRODUCTION

Dose rate was recognized as an important factor for biological effectiveness approximately 60 years ago, and dose rate effects were suggested to be associated with the oxygen concentration in living cells.^1^, ^2^ Specifically, it was proposed that living cells were spared due to intracellular oxygen depletion when electron beams were delivered at a high dose rate. The sparing effects induced by high‐dose‐rate irradiation did not attract much attention at that time, as research and development were primarily focused on maximizing therapeutic effectiveness. Over time, dose rate effects have once again attracted interest.^3^ Radiotherapy for cancer performed at an ultra‐high dose rate (UHDR: > 40 Gy/s) has been known as FLASH radiotherapy because of its excellent ability to spare healthy tissue while maintaining treatment effectiveness.^3^, ^4^ Although the advantages of FLASH radiotherapy have been widely reported,^5^ the tissue‐sparing effects have not been consistently observed. To optimize the performance of FLASH radiotherapy, the mechanisms underlying sparing effects should be understood. Plausible mechanisms are, for instance, the reduction of oxygen concentration by instantaneous irradiation, leading to an intracellular transient hypoxic condition,^6^ reactions between reactive oxygen species (ROS) formed by neighboring different tracks, resulting in the decrease of indirect action,^7^, ^8^ and a modification of immune response that inhibits the cancer metastasis.^9^, ^10^ While each of these mechanisms has been discussed, the first two—oxygen depletion and intertrack chemical reactions—could be suitable for quantitative investigation through computer simulation.

Monte Carlo simulations can be used as a complementary technique to experiments to study the behaviors of individual charged particles and water radiolysis species with stochastic models over time; thereby, an in silico study could be suitable for understanding the intertrack effects among water radiolysis species induced under UHDR conditions. Indeed, several studies on Monte Carlo simulations under UHDR conditions have been performed.^11^, ^12^, ^13^, ^14^, ^15^, ^16^ Alanazi et al.^11^ simulated intertrack reactions from 300 MeV protons in air‐free water, finding that higher event density increased radical recombination, reducing radical yields while increasing molecular yields. Ramos–Mendez et al.^12^ performed water radiolysis simulations and found that intertrack reactions, enhanced by sequential proton irradiation, affected the G values of hydrated electrons and hydroxyl radicals. Other studies^14^, ^15^, ^16^ also conducted investigations on intertrack reactions and analyzed how the yields of molecular species depend on dose rate. Furthermore, Lai et al.^13^ analyzed dissolved oxygen consumption, confirming that it is unlikely to be depleted at a typical absorbed dose (10–40 Gy) used in UHDR experiments.

Water radiolysis simulations under UHDR conditions have long computation times because a large number of species must be tracked; accurate reproduction of the experimental setup is challenging. Therefore, molecule transportation processes have been simplified to reduce simulation times using alternative models, such as the independent reaction time (IRT) method^11^, ^12^ or the Gillespie algorithm.^14^, ^16^, ^17^ However, accurate spatial tracking of molecular interactions is essential, as intertrack reactions are fundamentally governed by spatial proximity among radiolysis species. These simplified approaches, while computationally efficient even on CPUs, cannot adequately capture the spatial dependence critical for mechanistic studies of intertrack effects on radiolysis species. Therefore, developing a high‐fidelity simulation platform for water radiolysis under UHDR conditions is crucial for elucidating the spatially dependent chemical processes driven by intertrack reactions.

In the present study, we have developed a simulation platform based on MPEXS2.1‐DNA^18^, ^19^, ^20^, ^21^ to build a computational framework for investigating intertrack effects on water radiolysis under UHDR conditions. By leveraging GPU‐accelerated step‐by‐step (SBS) molecular tracking, we achieved high spatial precision in tracking molecular positions during water radiolysis under UHDR conditions. This computational platform focuses specifically on water radiolysis simulations as a foundational step before investigating biological endpoints such as DNA damage and cellular responses. Developing accurate radiation chemistry models under UHDR conditions could provide the simulation framework needed for future biological mechanism studies on the FLASH effect. To validate our model, we tracked water radiolysis species generated during proton irradiation, varying dose rates from a conventional rate (0.02 Gy/s) to UHDR conditions (up to 500 Gy/s). We assessed dose rate dependence of yields for key water radiolysis species (hydroxyl radicals, hydrated electrons, and hydrogen peroxide) and compared our results with published experimental data.^22^, ^23^, ^24^, ^25^ Additionally, we evaluated temporal changes in dissolved oxygen concentration to investigate transient hypoxia under UHDR conditions.

## 2 MATERIALS AND METHODS

### 2.1 MPEXS2.1‐DNA

MPEXS2.1‐DNA^18^, ^19^, ^20^, ^21^ is a Monte Carlo simulation code that runs on NVIDIA GPU devices. It is based on Geant4‐DNA^16^, ^26^, ^27^, ^28^, ^29^ version 10.7 Patch‐4, an extension package of the Geant4 simulation toolkit,^30^, ^31^, ^32^ for simulating track structures and water radiolysis at the subcellular scale. All the physics and chemistry codes of Geant4‐DNA have been reimplemented in CUDA, with a reengineered data structure suitable for GPU processing. Radiation chemistry simulations within the MPEXS2.1‐DNA framework consist of multiscale simulations, including the physics stage (< 1 ps) and the chemistry stage (> 1 ps). The physics stage calculates the spatial distributions of energy loss by simulating electromagnetic interactions of charged particles following the physics models described elsewhere.^18^, ^19^, ^20^ Primary water radiolysis species, such as hydrated electrons and hydroxyl radicals, are generated via dissociative decays of ionized and excited water molecules. Then, water radiolysis simulations are performed in the chemistry stage. A SBS approach^20^, ^21^—hereafter referred to as the conventional SBS (CONV‐SBS) approach—is applied to track the behavior of each species and obtain precise information on molecular positions over time. MPEXS2.1‐DNA runs more than 7600 times faster than Geant4‐DNA, meaning that a single GPU delivers the computing performance of 7600 CPU cores.

In our recent study,^21^ we implemented an alternative SBS model based on the theory of the Green's function of the diffusion equation (GFDE) for second‐order reactions, called GFDE‐SBS, in the MPEXS2.1‐DNA framework to increase the accuracy and speed of water radiolysis simulations under ion irradiation. In this approach, molecular species generated via water radiolysis, including ROS (e.g., O^•−^, O_2_, O_2_^•−^, HO_2_^•^, HO_2_^−^), are tracked as point‐like objects, while dissolved molecules (e.g., scavengers) are handled as spatially distributed species following first‐order or pseudo‐first‐order reaction kinetics. This equation‐based model follows an exponential law for reaction probabilities; dissolved species do not require explicit representation as point‐like objects, which significantly reduces memory consumption. Furthermore, in GFDE‐SBS, fixed time steps are applied for molecular transportation processes, allowing a constant number of iterations regardless of the increasing molecule number, thereby reducing computational times. Indeed, the GFDE‐SBS model is more than 10 times faster than the CONV‐SBS approach. This efficiency is crucial under UHDR conditions where a large number of species must be tracked. Thus, due to these advantages, we applied the GFDE‐SBS model for water radiolysis simulations under UHDR conditions in the present study.

### 2.2 Water radiolysis simulations under UHDR conditions

To develop a computational framework for investigating intertrack reactions among water radiolysis species generated under UHDR conditions, we developed a comprehensive simulation model based on MPEXS2.1‐DNA. This platform enables precise tracking of water radiolysis species under continuous proton irradiation by employing a SBS. The following setup was designed and validated through comparison with available experimental data.

**FIGURE 1:**
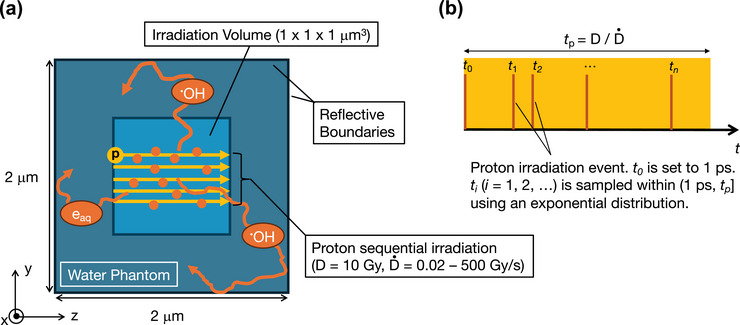
(a) Settings for water radiolysis simulations under UHDR conditions. (b) Sequential proton irradiation. A yellow rectangle shows the duration (*t_p_*), which is derived from the total absorbed dose (*D*) and dose rate ($\dot{D}$). Vertical orange bars indicate the times of proton irradiation events (*t_i_*, *i* = 0, 1, 2, …), which were sampled using an exponential distribution within the range of (1 ps, t_p_].

A 2 × 2 × 2 µm^3^ cubic phantom filled with liquid water at neutral pH was constructed (Figure 1a). A 1 × 1 × 1 µm^3^ volume was defined at the center of the phantom, continuously irradiated with protons. The incident energy of the protons was set to 55 MeV, representing a validated intermediate energy from experimental UHDR studies^22^, ^23^, ^24^, ^25^ that employed 27.5, 55, 68, and 230 MeV protons. This choice balances the availability of experimental validation in the low‐LET proton range—where observed dose rate trends are governed primarily by intertrack encounter probabilities and the modest LET variations are relatively minor—with the technical constraints of MPEXS2.1‐DNA (limited to protons up to 100 MeV). The irradiation positions of individual protons were randomly sampled on the xy boundary plane of the irradiation volume using uniform random numbers. Protons traveled along the z‐axis, and their tracking was terminated upon reaching the opposite boundary of the irradiation volume. The processes for secondary electrons generated via proton interactions were also stopped when leaving the irradiation volume. Note that the size of the phantom and the irradiation field were determined with reference to previous simulation studies.^11^, ^12^, ^13^

In physics simulations, the proton irradiation was repeated until the absorbed dose within the irradiation volume exceeded the predefined threshold of *D* = 10 Gy, which was a representative dose for UHDR experiments.^33^, ^34^ Each irradiation time point was randomly sampled based on an exponential distribution within a given duration (Figure 1b). Here, we simulated changes in yields of water radiolysis species over a wide range of dose rates: $\dot{D}$ = 0.02, 0.1, 0.3, 0.5, 1, 5, 10, 30, 50, 75, 100, 200, and 500 Gy/s. A duration ($t_{p}$) of sequential proton irradiation for individual cases was derived as $t_{p}=D/\dot{D}$. In general, a total absorbed dose is estimated by $D=N_{p}/A\times \mathrm{LET}/\rho$, where *N*_p_ is the proton number, *A* is the surface area (1 µm^2^), LET is the linear energy transfer for 55 MeV protons (1.169 keV/µm computed by the SRIM code^35^), and ρ is the water density (1 g/cm^3^). Thus, the proton number is derived from $N_{p}=DA\rho /\mathrm{LET}$, indicating that approximately 54 protons are required to irradiate the target volume in order to deliver an absorbed dose of 10 Gy. On the other hand, in our simulations, each sequential proton irradiation consisted of an average of 71 proton irradiation events, slightly more than the analytically calculated value. This discrepancy occurred because some protons were delivered near the edge of the irradiation volume and could pass through the volume without depositing significant energy. To ensure statistical reliability, we simulated 1000 independent sequential proton irradiation scenarios for each dose rate condition.

In chemistry simulations, the chemical reactions of each water radiolysis species were tracked for 1000 s using the GFDE‐SBS model. A comprehensive list of the molecular species, along with all reactions and rate constants considered in this study, is provided in the Supporting Information (see Section S1). To gain insight into oxygen depletion under UHDR conditions, we quantified the yields of water radiolysis species under oxygenated conditions. The concentration of dissolved oxygen in percentage $pO_{2}$ is the ratio of partial oxygen pressure in liquid water to the atmospheric pressure *P*_atm_ (760 mmHg). Thus, $pO_{2}$ can be expressed in mmHg by $pO_{2}\;\left[\mathrm{mmHg}\right]=pO_{2}\;\left[\%\right]\times P_{\mathrm{atm}}$. Additionally, we convert $pO_{2}$ in µM by $pO_{2}\;\left[\mu M\right]=pO_{2}\;\left[\mathrm{mmHg}\right]\times H_{c}$, where *H*_c_ (1.26 µM/mmHg) is the coefficient of Henry's Law for dissolved oxygen in liquid water.^13^, ^36^ We set the concentration of dissolved oxygen to $pO_{2}=25\%$ (239.4 µM) for the oxygenated conditions to match the experimental conditions^22^, ^24^, ^25^ used in our validation studies, while $pO_{2}=0\%$ for the deoxygenated conditions. This choice enables direct quantitative comparison between our simulation results and experimental data,^22^, ^24^, ^25^ essential for model validation. We handled the following chemical reactions involving dissolved oxygen distributed homogeneously as pseudo‐first‐order reactions:

(R1) $$H^{•}+O_{2}\to {\mathrm{HO}}_{2}^{•}\;\left(k=1.27\times 10^{10}\;\left[M^{-1}s^{-1}\right]\right),$$

(R2) $$e_{\mathrm{aq}}^{-}+O_{2}\to O_{2}^{•-}\;\left(k=1.48\times 10^{10}\;\left[M^{-1}s^{-1}\right]\right).$$

The reaction probability of a species interacting with dissolved oxygen during time‐stepping Δt is given by $p=1-\exp(-k•pO_{2}\left[\mu M\right]•\Delta t)$, where *k* is a reaction rate constant in M^−1^s^−1^, so that the coefficient $k•pO_{2}\left[\mu M\right]$ is regarded as a scavenger power, which has a unit of s^−1^. Continuous proton irradiation of the irradiation volume induced the delivery of hydrated electrons ($e_{\mathrm{aq}}^{-}$) and hydrogen radicals (H^•^), and the concentration of dissolved oxygen was significantly changed via reactions R1 and R2. Thus, at each time step, the number of oxygen generation and consumption events was recorded and used to update the spatially averaged oxygen concentration across the entire irradiation volume. This approach enabled the temporal tracking of oxygen dynamics under both oxygenated and deoxygenated conditions.

The simulation handles radiolysis species generated by sequential proton irradiation events as follows: each proton generates its own set of radiolysis species at a specific time point, and these species continue to diffuse and react throughout the entire simulation duration (up to 1000 s). When a new proton is delivered, the simulation kernel resets the time step size, which is predetermined (see Section S2), to the initial value to accurately capture the rapid intratrack reactions, and then progressively increases the time step to 1000 s. The newly generated species from subsequent protons coexist with previously generated species that remain in the simulation volume. This approach allows us to track intertrack reactions that occur naturally when species originating from different proton events encounter each other spatially during their diffusion processes. This capability represents a fundamental advantage of our GPU‐based SBS approach over alternative methods such as IRT, which cannot accurately track the precise molecular positions required for realistic intertrack reaction modeling. This spatial precision is essential for accurately modeling the enhanced radical‐radical interactions characteristic of UHDR conditions, where the high density of radiolysis species makes intertrack effects dominant. Additionally, this computationally intensive tracking can be performed within a reasonable computational time owing to the ultra‐parallel processing capability of GPU devices.

Due to the long duration of 1000 s, some water radiolysis species were expected to reach the boundary of the water phantom repeatedly. Thus, we applied a reflective condition to the box boundaries. When a water radiolysis species reached a box boundary, it was reflected and its direction of motion was changed in the manner described in.^17^, ^37^ The efficacy of the reflective boundary condition was verified by comparing the time evolution of the number of water radiolysis species simulated by MPEXS2.1‐DNA with the analytical solution. Details are described in the Supporting Information (see Section S2).

### 2.3 Validation for water radiolysis simulations in UHDR conditions

We validated the simulation model for water radiolysis under UHDR conditions from the following three perspectives: (1) time evolution of radiation chemical yields (G values, species/100 eV) of molecular species, (2) dose rate dependence of G values, and (3) temporal changes in the concentration of dissolved oxygen.

#### 2.3.1 Time evolution of G values of molecular species

We visualized the four‐dimensional evolution (x, y, z, and time) of water radiolysis species during continuous proton irradiation, clearly illustrating the diffusion and chemical reactions of each species. Subsequently, the temporal change in radiation chemical yield (G value), defined as the number of molecules produced or lost per 100 eV of deposited energy, was calculated for hydroxyl radicals. As expected, the yield increased during proton irradiation.

#### 2.3.2 Dose rate dependence of G values of molecular species

The dose rate dependence of the G values of hydroxyl radicals was computed under UHDR conditions, and the results were compared with experimental data.^22^, ^24^, ^25^ Furthermore, G values of other species, including hydrated electrons and hydrogen peroxide, were also computed and compared with published data^23^, ^25^, ^38^ to assess the accuracy of our simulation framework.

#### 2.3.3 Temporal change in dissolved oxygen concentration

Previous experimental studies^24^, ^39^, ^40^ have reported that while oxygen depletion occurs under UHDR conditions, the extent of depletion at a typical UHDR dose (10–40 Gy) is insufficient to cause complete oxygen depletion. This observation was also supported by another simulation study.^13^ However, the rate of oxygen consumption and its dose rate dependence remain topics of active investigation. As depicted in Figure 1a, the concentration of dissolved oxygen in the irradiation volume significantly changed during sequential proton irradiation through reactions R1 and R2. Thus, we analyzed the temporal variation in oxygen concentration to quantify the depletion rate per absorbed dose of 1 Gy and to compare our results with experimental trends reported elsewhere.^24^, ^39^, ^40^

## 3 RESULTS

### 3.1 Time evolution of G values for water radiolysis species

#### 3.1.1 4‐dimensional evolution of water radiolysis species

**FIGURE 2:**
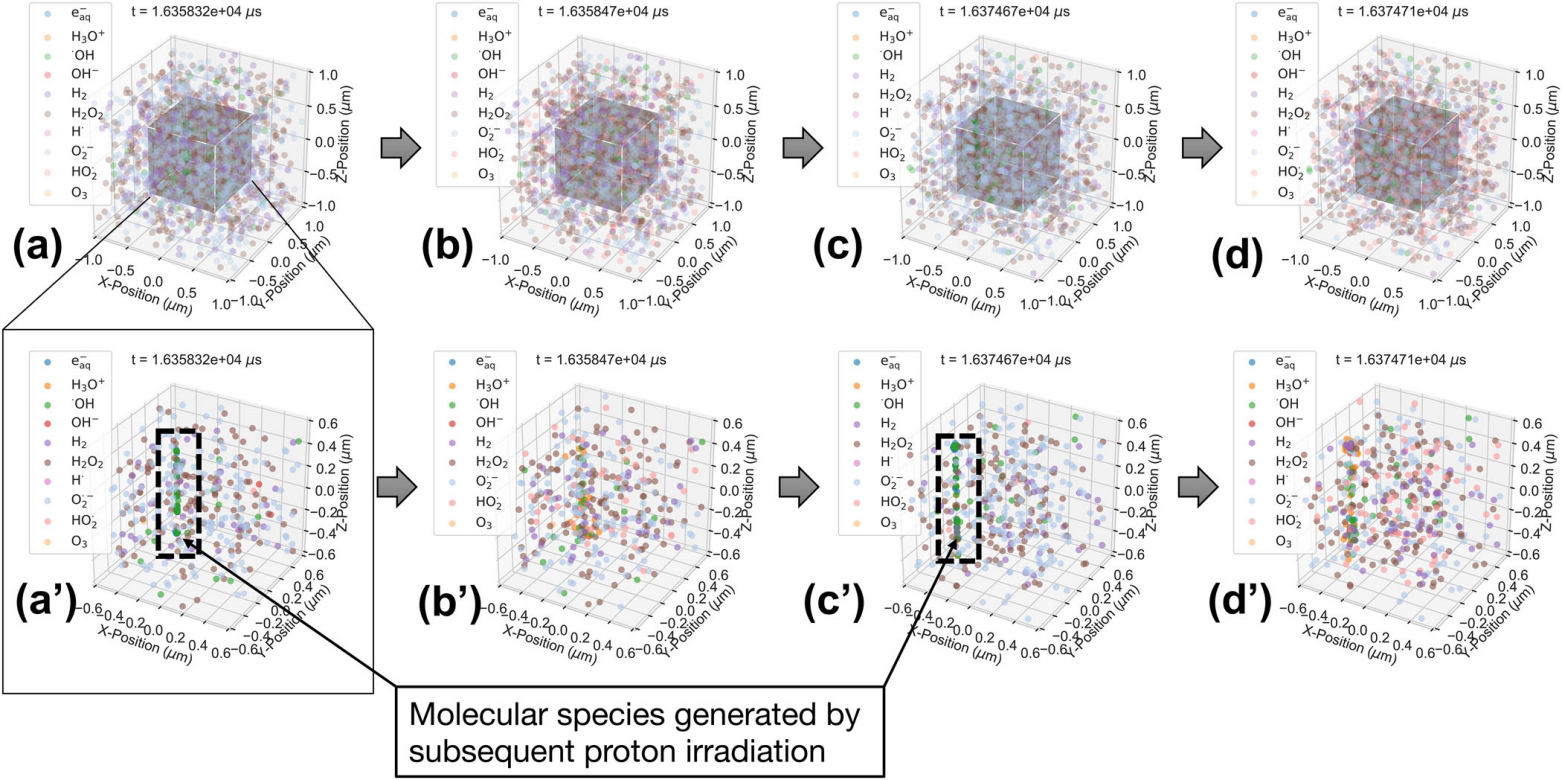
Molecular diffusion and chemical reactions with neighboring molecules under sequential irradiation with the 55 MeV proton beam at a UHDR of 500 Gy/s, observed at (a) *t* = 16.36 ms, and at (b) 0.15 µs, (c) 16.35 µs, and (d) 16.39 µs following (a). A cube at the center represents the irradiation volume, with enlarged views shown in (a′–d′). The two dashed boxes in (a’) and (c’) show the molecular species generated by subsequent proton irradiation events.

To visually demonstrate the diffusion and chemical reactions for each species during continuous proton irradiation, we evaluated the 4‐dimensional evolution (x, y, z positions and time) of water radiolysis species (Figure 2). This figure shows the spatiotemporal changes in water radiolysis species generated under 55 MeV protons at a dose rate of 500 Gy/s. Protons were continuously delivered until the absorbed dose to the irradiation volume reached 10 Gy. Along with the trajectories of the primary proton beams and secondary electrons, radiolysis species were generated through ionization, excitation, and dissociative attachment processes. Over time, these species diffused and underwent reactions with other nearby species (Figure 2a–d). The two dashed‐line boxes in Figure 2a and c highlight the radiolysis species produced by the subsequent proton irradiation events. These species diffused and gradually reacted with those generated by earlier events, indicating that intertrack reactions between radiolysis species occurred under UHDR conditions.

#### 3.1.2 Time dependence of G values of hydroxyl radicals

**FIGURE 3:**
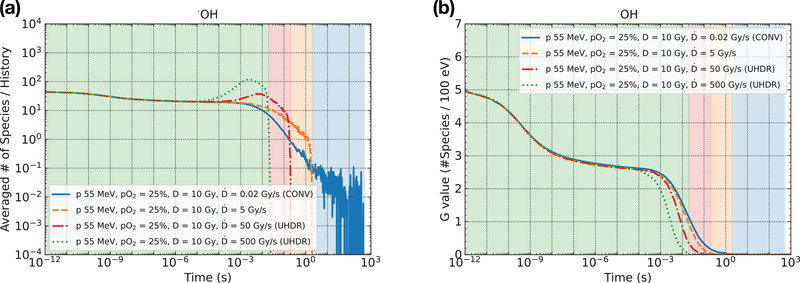
Changes in the (a) mean number and (b) G value of hydroxyl radicals over time under the 55 MeV proton beam with varying dose rates: blue solid line, 0.02 Gy/s; orange dashed line, 5 Gy/s; red dash‐dotted line, 50 Gy/s; and green dotted line, 500 Gy/s. The shaded regions represent the durations of pulse beam irradiation: blue for 0.02 Gy/s (*t*_p_ = 500 s), orange for 5 Gy/s (*t*_p_ = 2 s), red for 50 Gy/s (*t*_p_ = 200 ms), and green for 500 Gy/s (*t*_p_ = 20 ms).

We investigated the temporal evolution of the mean numbers of hydroxyl radicals from the initial irradiation (1 ps) with 55 MeV protons to the end of the simulation (1000 s) at four dose rates: 0.02, 5, 50, and 500 Gy/s (Figure 3a). Here, 0.02 Gy/s represents a conventional dose rate, whereas 50 and 500 Gy/s correspond to UHDR conditions. As protons were continuously delivered, the number of hydroxyl radicals increased. At 500 Gy/s, protons were delivered instantaneously compared to 0.02 Gy/s, causing the number of hydroxyl radicals to rise rapidly in the earlier time region (10^−5^ to 10^−2^ s), resulting in a dense distribution of hydroxyl radicals and other water radiolysis products. The dense distribution of water radiolysis species enhanced intertrack reactions formed by different tracks in the vicinity, leading to the reduction of hydroxyl radicals on a later time scale.

The time dependence of the radiation chemical yield for hydroxyl radicals at each dose rate case was also calculated (Figure 3b). With increasing dose rates, water radiolysis species were more densely distributed immediately after proton delivery by the pulse beam, promoting intertrack reactions. Consequently, the G values of hydroxyl radicals in the high‐dose‐rate cases (50 and 500 Gy/s) decreased more rapidly than in the conventional case (0.02 Gy/s). Our findings agree with the results of previous simulation studies.^11^, ^12^, ^13^, ^14^, ^15^, ^16^

### 3.2 Dose rate dependence of G values for water radiolysis species

#### 3.2.1 Hydroxyl radicals

**FIGURE 4:**
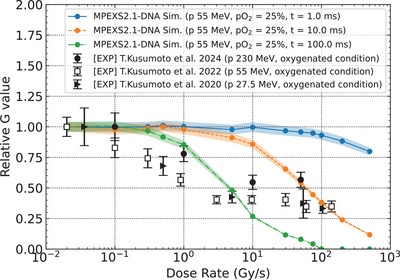
Simulation results of the dose rate dependence of the relative G values of hydroxyl radicals under the 55 MeV proton irradiation in oxygenated conditions ($pO_{2}=25\%$); the blue solid, orange dashed, and green dash‐dotted lines represent the values calculated at 1, 10, and 100 ms after the initial proton delivery. The shaded region indicates ± 4% of the relative G value as the range of the standard deviation for hydroxyl radicals. The relative G values of 7OH‐C3CA measured under oxygenated conditions in previous studies (filled triangles,^22^ open squares,^24^ and filled circles^25^) and associated errors are also presented.

The dose rate dependence of the relative G values of hydroxyl radicals in neutral pH water (pH = 7) with oxygenated conditions ($pO_{2}=25\%$) was evaluated at 1, 10, and 100 ms. These time points were chosen to capture the temporal evolution of the G values of hydroxyl radicals, which are consumed rapidly through radical‐radical reactions under UHDR. Relative G values were calculated by normalizing the G value at each dose rate (from 0.02 Gy/s to 500 Gy/s) to the value obtained at the lowest dose rate of 0.02 Gy/s. The standard deviation of the calculated G values of hydroxyl radicals was estimated to be less than 2%; therefore, bands representing a ± 4% range are shown for better visual clarity. The relative G values decreased monotonically with increasing dose rate. Over time, most hydroxyl radicals were consumed, and the relative G values approached zero, particularly at higher dose rates (Figure 4). The substantial reduction in the G values of hydroxyl radicals is likely due to intertrack reactions induced under UHDR conditions. The yield of each reaction involving hydroxyl radicals increased rapidly with increasing dose rate, eventually becoming saturated (see Section S3.1 in the Supporting Information).

This trend is consistent with experimental results,^22^, ^24^, ^25^ in which the dose rate dependence of the relative G values of 7‐hydroxy‐coumarin‐3‐carboxylic acid (7OH‐C3CA) was measured. The experimental relative G values of 7OH‐C3CA were obtained by normalizing the values at each dose rate to that at the lowest dose rate (i.e., 0.02 Gy/s), similar to our simulation approach for hydroxyl radicals. 7OH‐C3CA is produced through the reaction of hydroxyl radicals with coumarin‐3‐carboxylic acid (C3CA), which is widely used as a hydroxyl radical scavenger. It should be noted that direct measurement of hydroxyl radicals is challenging; thus, molecular probes such as C3CA are commonly employed for indirect detection. Notably, while experimental measurements rely on secondary products such as 7OH‐C3CA, which are formed through radical‐radical interactions, for indirect estimation of hydroxyl radicals, our simulations directly calculate the G values of hydroxyl radicals themselves. Therefore, the observed differences between experimental and simulation results, as shown in Figure 4, are reasonable. Despite differences in how the G values of hydroxyl radicals were evaluated, both approaches reveal the same underlying mechanism. Specifically, enhanced intertrack reactions at higher dose rates led to reduced effective hydroxyl radical availability. This consistency supports the validity of our computational approach for investigating water radiolysis under UHDR conditions.

#### 3.2.2 Hydrated electrons

**FIGURE 5:**
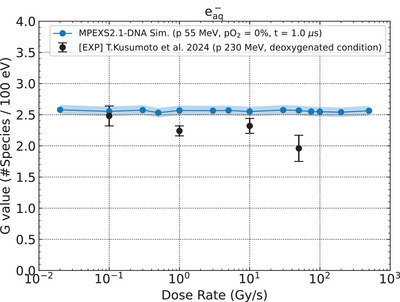
G values of hydrated electrons as a function of dose rate calculated at 1 µs after the initial irradiation with the 55 MeV proton beam under deoxygenated conditions (blue solid line) and experimental data (filled circles).^25^ The shaded area indicates the standard deviation of the calculated G values of hydrated electrons, which is within ± 3% of the mean.

Since hydrated electrons efficiently react with dissolved oxygen (R2), the G values of hydrated electrons are normally measured under deoxygenated conditions in experiments. In the present study, we calculated the G values of hydrated electrons at 1 µs after irradiation, reflecting the typical experimental measurement timeframe for these species under deoxygenated conditions. The G values of hydrated electrons, calculated under the deoxygenated conditions ($pO_{2}=0\%$), are shown as a function of dose rates ranging from 0.02 to 500 Gy/s (Figure 5). The standard deviation of the calculated G values of hydrated electrons was estimated to be less than 1.5%; therefore, a ± 3% range is shown to enhance visual recognition in the figure. We did not observe significant changes in the G values of hydrated electrons at different dose rates, and the results were consistent with previous simulations.^12^, ^15^ Overall, our simulation results agree with the experimental data^25^ under 230 MeV proton irradiation. However, a discrepancy was observed around 50 Gy/s, where experimental data suggested a decrease while our simulations showed constant yields. This difference could be attributed to competitive reactions not fully captured in our current model, as discussed in Section 4.1.

#### 3.2.3 Hydrogen peroxide

**FIGURE 6:**
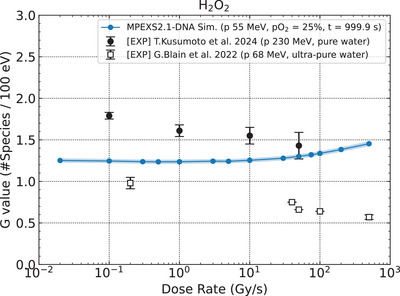
The dose rate dependence of G(H_2_O_2_) at 1000 s after the initial irradiation with the 55 MeV proton beam under oxygenated conditions. The shaded region shows the standard deviation of the calculated G values within ± 2% of the mean. The filled circles^25^ and the open squares^23^ represent experimental data.

We calculated the dose rate dependence of G values of hydrogen peroxide, G(H_2_O_2_), following 55 MeV proton irradiation at the end of simulation (1000 s) under oxygenated conditions $(pO_{2}$ = 25%) (Figure 6). As H_2_O_2_ is a stable end‐product that accumulates over time mainly through radical‐radical recombination reactions, we evaluated the G(H_2_O_2_) at the simulation end time. The standard deviation of the calculated G(H_2_O_2_) was estimated to be less than 1%; therefore, a ± 2% range is shown for better visual recognition. As the dose rate increased, the G value of H_2_O_2_ increased monotonically because radical‐radical reactions, mainly:

(R3)

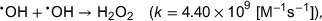

were enhanced among different proton tracks in the vicinity. The trend indicated by our simulations agreed with the previous simulation studies.^11^, ^14^, ^15^

While our simulation results showed a 20% increasing trend in hydrogen peroxide yield with increasing dose rate, previous experimental studies^23^, ^25^ have reported the opposite: a gradual decrease (20%–40%) in G(H_2_O_2_) under UHDR conditions. The observed decrease in hydrogen peroxide yields with increasing dose rate could be attributed to the enhanced consumption of hydroxyl radicals through their reactions with other species (R4‐R6):

(R4)

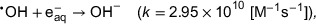



(R5)

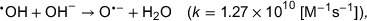



(R6)

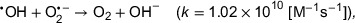

which was discussed elsewhere.^38^ In other words, as the dose rate increases, hydroxyl radicals—precursors to hydrogen peroxide—are more likely to undergo alternative reactions, thereby reducing the G(H_2_O_2_). In our simulations, as described in the supporting information (see Section S3.2), the yields of R4 and R5 showed no differences among dose rate cases. In comparison, regarding R6, the yields became larger at lower dose rates, reducing the number of precursors for H_2_O_2_ production (i.e., hydroxyl radicals). As a result, we observed that the calculated G(H_2_O_2_) increased with increasing dose rates. Notably, this increasing trend contradicts experimental observations reporting a 20%–40% decrease in G(H_2_O_2_) under UHDR conditions. This fundamental discrepancy suggests that our current reaction set could be missing key competitive pathways that become significant under UHDR conditions, as discussed in Section 4.1.

### 3.3 Changes in the dissolved oxygen concentration over time

**FIGURE 7:**
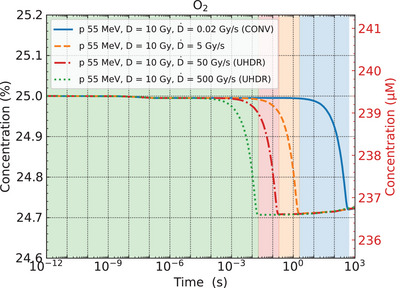
Simulated oxygen concentrations as a function of time during proton irradiation at 0.02 Gy/s (blue solid line), 5 Gy/s (orange dashed line), 50 Gy/s (red dash‐dotted line), and 500 Gy/s (green dotted line). The shaded region for each curve shows the pulse duration: blue for 0.02 Gy/s (*t*_p_ = 500 s), orange for 5 Gy/s (*t*_p_ = 2 s), red for 50 Gy/s (*t*_p_ = 200 ms), and green for 500 Gy/s (*t*_p_ = 20 ms).

The changes in the dissolved oxygen concentration in the irradiation volume over time were calculated at four dose rates (0.02, 5, 50, and 500 Gy/s) to provide insight into the concept of oxygen depletion (Figure 7). The initial concentration of dissolved oxygen was set to $pO_{2}=25\%$ (239.4 µM). Sequential proton irradiation activated reactions R1 and R2, leading to a gradual depletion of oxygen concentration, with the lowest concentration reached at the end of each irradiation period.

**FIGURE 8:**
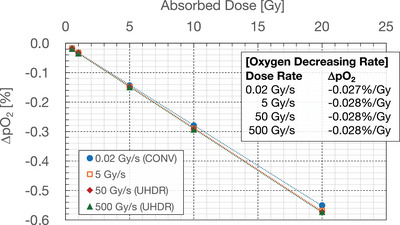
Decrease in dissolved oxygen concentration as a function of absorbed dose (0.5–20 Gy) for each dose rate: 0.02 Gy/s (blue filled circles), 5 Gy/s (orange open squares), 50 Gy/s (red filled diamonds), and 500 Gy/s (green filled triangles). Oxygen depletion rates per 1 Gy were computed for each dose rate case by linear interpolation.

Extending the absorbed dose range from 0.5 to 20 Gy, we evaluated the oxygen depletion rate per absorbed dose of 1 Gy (Figure 8), which was derived as approximately 0.028%/Gy (0.27 µM/Gy). The dose rate dependence of the oxygen depletion rate was negligible in our simulation. Kusumoto et al.^24^ directly measured the depletion rate of oxygen concentration in oxygenated neutral pH water under 0.02 Gy/s and 80 Gy/s (UHDR) proton irradiation, reporting 0.27 µM/Gy without dose rate dependence. Additionally, Cao et al.^39^ quantified oxygen depletion under electron UHDR irradiation with a BSA solution, reporting a depletion rate of 0.28–0.30 µM/Gy. Jansen et al.^40^ reported that the amount of oxygen depletion in water per absorbed dose of 10 Gy under UHDR conditions with photons, protons, and carbon ions was in the range of 0.04%–0.25%, corresponding to 0.004%–0.025%/Gy. While the dose rate dependence of oxygen depletion was negligible in our simulations, their studies^39^, ^40^ reported that oxygen depletion rates decreased with increasing dose rate, indicating a discrepancy that could be related to missing competitive reactions discussed in Section 4.1. Nevertheless, our simulation results generally agree with these experimental results. Notably, despite differences in experimental conditions (protons vs. electrons, pure water vs. BSA solution), the consistent oxygen depletion rates across different studies validate the fundamental chemical mechanisms (R1 and R2) implemented in our model.

Based on our simulation results, we estimated the absorbed dose required for complete oxygen depletion. When $pO_{2}=25\%$, the total absorbed dose required for the depletion of dissolved oxygen was derived to be approximately 900 Gy. A previous Monte Carlo‐based study^13^ also estimated the required dose of oxygen depletion to be in the range of 100–1,000 Gy, depending on the initial oxygen concentration in neutral pH water. These calculated doses associated with transient hypoxia are much higher than the typical doses of 10–40 Gy in UHDR experiments.^33^, ^34^ Thus, complete oxygen depletion is unlikely under typical UHDR conditions. However, as shown in Figure 7, oxygen was consumed more rapidly under UHDR conditions compared to the conventional condition (0.02 Gy/s). This finding suggests that local oxygen depletion could occur in regions of high dose deposition. In a future study, we plan to extend this model by introducing spatial grid segmentation in the irradiation volume to evaluate local oxygen gradients and better describe heterogeneous oxygen depletion.

## 4 DISCUSSION

### 4.1 Missing competitive reactions affecting molecular yields under UHDR conditions

As shown in Figure 6, the gradual decrease in the G(H_2_O_2_) measured under UHDR conditions could not be reproduced in our present model. This discrepancy suggests that a competitive reaction preventing the recombination of hydroxyl radicals may be missing. Zhang et al.^38^ proposed the following third‐order reaction:

(RE1)

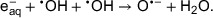




While RE1 can be formally expressed as a combination of R4 and R5, both of which showed no clear dose rate dependence in our simulations (see Section S3.2), the authors^38^ suggested that it may also be treated as an effective third‐order process with a rate proportional to 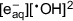. This reaction could compete with R3, which is a second‐order process with rate 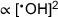, when

(1) $$k_{\mathrm{RE}1}\left[e_{\mathrm{aq}}^{-}\right]{{[}^{•}\mathrm{OH}]}^{2}≳k_{R3}{{[}^{•}\mathrm{OH}]}^{2}$$

Here, *k*_R3_ [M^−1^s^−1^] and *k_RE1_* [M^−2^s^−1^] denote the reaction rate constants of R3 and RE1, respectively. The inequality (1) defines a critical hydrated electron concentration above which RE1 becomes competitive:

(2) $$\left[e_{\mathrm{aq}}^{-}\right]≳\frac{k_{R3}}{k_{\mathrm{RE}1}}$$

Because of this higher‐order dependence, the contribution of RE1 could not be significant under conventional conditions, where the concentration of hydrated electrons remain far below the threshold (2). In comparison, under UHDR conditions—where the concentrations of hydroxyl radicals and hydrated electrons instantaneously and transiently increase—this threshold could be exceeded, rendering RE1 a potentially significant additional pathway. In this context, R4 represents a two‐body reaction occurring within an isolated track, whereas RE1 effectively describes situations under dense track conditions where radicals from different tracks coexist before diffusing away. From this viewpoint, RE1 is not merely equivalent to R4 and R5, but rather an additional competitive pathway that could contribute under UHDR conditions. To be consistent with the measured G(H_2_O_2_), it could be necessary to take this third‐order reaction into account as a possible competitor to the recombination of hydroxyl radicals.

Furthermore, incorporating RE1 could also reproduce the dose rate dependence of oxygen depletion rates. Previous experimental studies^39^, ^40^ reported that oxygen depletion rates decreased with increasing dose rate, while our simulations showed no significant dose rate dependence (Figure 8). Under UHDR conditions, reaction RE1 could compete with R2 ($e_{\mathrm{aq}}^{-}+O_{2}\to O_{2}^{•-}$) for hydrated electrons, thereby reducing oxygen consumption at higher dose rates. This mechanism could explain the experimentally observed dose rate dependence on oxygen depletion that our current model fails to reproduce.

Additionally, the same competitive mechanism could help explain the dose rate dependence of the G values of hydrated electrons. While our current simulations show relatively constant yields across dose rates (Figure 5), experimental data suggest a decrease at higher dose rates. As RE1 consumes hydrated electrons in competition with other reactions, including R2, its incorporation could improve agreement with the experimental trend of decreasing G values of hydrated electrons under UHDR conditions.

The current GFDE‐SBS model^21^ of MPEXS2.1‐DNA calculates reaction probabilities based on the diffusion‐controlled encounter of two molecular species, determining whether they approach within the reaction radius during each time step. Extending this framework to third‐order reactions, such as RE1, requires calculating the probability of simultaneous three‐body encounters, for which, to the best of our knowledge, no established theoretical model exists for water radiolysis using a Monte Carlo method. A fundamental algorithmic development is needed, including theoretical work and validation beyond parameter adjustment. Therefore, we position the incorporation of third‐order reactions as a critical future development rather than an immediate extension of the current work.

### 4.2 Computational performance for water radiolysis under UHDR conditions

To demonstrate the computational efficiency and scalability of our GPU‐based simulation platform for water radiolysis under UHDR conditions, we performed comprehensive benchmark calculations by varying the number of independent sequential irradiation scenarios. These scenarios, ranging from 500 to 5000, were processed concurrently on a single GPU, enabling large‐scale statistical analysis. In each scenario, protons were continuously delivered until a total absorbed dose of 10 Gy was reached at a dose rate of 500 Gy/s, corresponding to an average of 71 proton irradiation events. An average of 9016 water radiolysis species were cumulatively generated over the course of each simulation scenario and subsequently tracked for 1000 s. The key advantage of our approach is that thousands of these independent scenarios can be processed simultaneously, with each scenario representing a water radiolysis process under identical UHDR conditions. The GPU device used in this benchmark was an NVIDIA^®^ RTX™ 6000 Ada Generation with 48 GB memory capacity.

Table 1 summarizes the benchmark results under different numbers of parallel scenarios. As the number of parallel scenarios increased, the processing time scaled almost linearly, yielding a stable throughput of 1114–1154 scenarios per hour. This consistency indicates that the overhead associated with parallelization was minimal. In addition to the near‐linear scaling of processing performance, memory usage also increased almost linearly with the number of molecular species as the number of parallel scenarios increased. As mentioned in Section 2.1, we treated reactions involving dissolved oxygen as pseudo‐first‐order reactions. This approach eliminates the need to represent dissolved species as point‐like objects, thereby enabling parallel processing of up to 5000 independent scenarios on a single GPU. In the case of 5000 scenarios processed in parallel, approximately 45 million molecular species were cumulatively simulated across all time steps, and the computation was completed in 4.3 h.

**TABLE 1:** Benchmark results for various numbers of independent scenarios in water radiolysis simulations under UHDR conditions.

Parallel Scenarios	Processing Time	The number of scenarios processed per hour	Cumulative total of species simulated	GPU Memory Usage
500	0.45 h	1114	4 507 566	5.8 GB
1000	0.89 h	1138	9 015 372	9.5 GB
3000	2.61 h	1149	27 045 955	24.2 GB
5000	4.33 h	1154	45 078 923	39.2 GB

These benchmark results demonstrate that even for computationally intensive simulations under UHDR conditions, our simulation platform can obtain statistically meaningful results within a practical timeframe. Furthermore, the computational scale can be further expanded because it supports multi‐GPU environments. Additionally, it enables comprehensive parameter space exploration related to UHDR conditions that are feasible within practical timeframes.

### 4.3 Current limitations

The present study focuses on water radiolysis in pure aqueous systems without cellular scavengers, representing a fundamental limitation for direct biological interpretation. In cellular environments, numerous biomolecules (e.g., glutathione) compete for radical species, altering the radical yields observed in our simulations.

The absence of cellular scavengers in our model likely overestimates the availability of ROS compared to biological conditions. However, this simplified system provides essential baseline data for understanding the fundamental radiation chemistry processes under UHDR conditions. Future extensions of this work will incorporate cellular scavengers to bridge the gap between pure water radiolysis and biological systems.

### 4.4 Future prospects

The present study provides a radiation chemistry foundation that can serve as a starting point for future biological modeling. While DNA damage and cellular responses are beyond the scope of this work, the accurate simulation of radical species distributions and yields is a necessary step for such investigations.

In future work, this computational platform will be extended to different particle types (e.g., electrons, photons, and carbon ions), enabling a quantitative investigation of intertrack reactions under UHDR conditions. Furthermore, the theoretical framework for incorporating third‐order reactions discussed in Section 4.1 will be developed to enhance the model accuracy. Such comparisons are important to understand how track structure affects radical‐radical interactions relevant to the G values of hydroxyl radicals and whether the enhanced intertrack reactions observed in the present study occur with other particle types as well. Additionally, we will assess the influence of beam parameters on intertrack reactions relevant to the G values of hydroxyl radicals, drawing on a previous study.^41^ The parameters to be considered—such as irradiation field size, pulse duration, and pulse repetition—are known to have complex interrelationships.

Building on the assessment of beam parameters, the next stage will involve biological modeling. The DNA double‐helix structure will be incorporated into the SBS approach to analyze the damage resulting from interactions between radicals and DNA molecules. Specifically, using the 3D distribution data simulated (Figure 2), we aim to calculate the probabilities of single‐strand breaks (SSBs) and double‐strand breaks (DSBs), as well as model cell survival rates and DNA repair efficiency. This biological model will be implemented following a previous study.^42^ The strand breaks induced under UHDR conditions will be quantitatively analyzed utilizing our MPEXS2.1‐DNA simulation method, with reference to a previous experimental study.^43^ This approach is expected to help clarify the processes underlying the reduced damage to normal tissues, which is a key mechanism of the FLASH effect under UHDR conditions.

## 5 CONCLUSION

The present study developed a GPU‐based computational framework for water radiolysis simulations under UHDR conditions using MPEXS2.1‐DNA. Our SBS approach enabled precise spatiotemporal tracking of intertrack reactions, beyond the capability of conventional simplified methods. Additionally, we demonstrated that large‐scale statistical analysis is computationally feasible within practical timeframes, which represents a significant advantage for investigating the enhanced radical‐radical interactions in UHDR conditions.

In the present study, we found that G values of hydroxyl radicals decreased with increasing dose rate due to enhanced radical–radical interactions between neighboring tracks, and eventually plateaued at values near zero in the UHDR region, matching experimental trends. For hydrated electrons under oxygen‐free conditions, no significant dose rate dependence was found. In comparison, the G(H_2_O_2_) increased with the dose rate in our simulations, differing from experimental observations that showed a decline, likely due to the absence of competitive reactions in our model, which we plan to address.

We also assessed oxygen consumption under UHDR conditions. Our simulations showed continuous proton irradiation reduced dissolved oxygen, consistent with experimental measurements across different conditions (e.g., protons vs. electrons, pure water vs. BSA solution). While complete oxygen depletion was unlikely at typical UHDR doses (10–40 Gy), our results suggest that local oxygen depletion could occur in regions of high dose deposition.

Despite these strengths, the current model did not reproduce the experimentally observed decrease in the G(H_2_O_2_), indicating that some competitive reaction pathways could be missing. Therefore, while the framework provides a suitable basis for mechanistic studies of UHDR‐induced radical–radical interactions, further refinement is required before it can fully reproduce all experimental observables.

In future work, we plan to extend our framework to other particles (e.g., electrons, photons, carbon ions), integrate cellular‐scale geometry such as DNA structures, and quantify DNA damage. These developments are expected to deepen our in silico understanding of radiochemical dynamics and contribute to unraveling the mechanism of the FLASH effect.

## CONFLICT OF INTEREST STATEMENT

The authors have no relevant conflicts of interest to disclose.

## DECLARATION OF GENERATIVE AI TECHNOLOGY IN THE WRITING PROCESS

The authors used GPT‐5 (OpenAI) in order to improve grammar and readability for the manuscript. No AI tools were used for data analysis, code generation, figure creation, or reference generation. All content was reviewed and verified by the authors, who take full responsibility for the manuscript.

## Supporting information



Supporting Information

## Data Availability

The datasets that support the findings of this study are available from the corresponding author upon reasonable request.
