# Supplementary material for: A GPU‐based Monte Carlo model for water radiolysis under ultra‐high dose rate irradiation: Development and validation with MPEXS2.1‐DNA
Source: Med Phys. 2025 Oct 24;52(11):e70071. doi: 10.1002/mp.70071 (PMC12551771; doi:10.1002/mp.70071)
Supplement: Supplementary file 1 — Supporting Information [file MP-52-0-s001.pdf]

Supporting information for "A GPU-based Monte Carlo model for water radiolysis under ultra-high dose rate irradiation: Development and validation with MPEXS2.1-DNA"

Shogo Okada[1]*, Koichi Murakami[1], Tamon Kusumoto[2], Katsuya Amako[1], and Takashi Sasaki[1]

1) *High Energy Accelerator Research Organization (KEK), 1-1, Oho, Tsukuba, Ibaraki, 305-0801, Japan*

2) *National Institutes for Quantum Science and Technology (QST), 4-9-1 Anagawa, Inage-ku, Chiba, 263-8555, Japan*

*Corresponding Author, Email: shogo.okada@kek.jp

S1. Molecular species and chemical reactions considered in the GFDE-SBS model for MPEXS2.1-DNA

The GFDE-SBS model of MPEXS2.1-DNA[1] employs the parameter set of the TRACIRT[2] code, including diffusion coefficients and radii for water radiolysis species (Table S1) and reaction rate constants of chemical reactions between two reactants (Table S2). The present study applied them.

Table S1. Molecular species handled in the GFDE-SBS model of MPEXS2.1-DNA, along with their diffusion coefficients and radii.

Species	Diffusion Coefficient [$\times10^{-9}$ m^2/s]	Radius [nm]	Species	Diffusion Coefficient [$\times10^{-9}$ m^2/s]	Radius [nm]
e^-_{aq}	4.9	0.5	$O_2^{\bullet-}$	1.75	0.22
H_3O^+	9.46	0.25	$HO_2^\bullet$	2.3	0.21
$H^\bullet$	7.0	0.19	HO_2^-	1.4	0.25
$^\bullet OH$	2.2	0.22	$O(^3P)$	2.0	0.2
H_2O_2	2.3	0.21	$O^{\bullet-}$	2.0	0.25
H_2	4.8	0.14	$O_3^{\bullet-}$	2.0	0.2
OH^-	5.3	0.33	O_3	2.0	2.0
O_2	2.4	0.17			

Table S2. Chemical reactions and their reaction rate constants (k_{obs}) considered in the GFDE-SBS model in MPEXS2.1-DNA. For first-order reactions, indicated by the symbol *, the value of k_{obs} is given in s^{-1}.

Index	Reaction	k_{obs} [$M^{-1}s^{-1}$]	Index	Reaction	k_{obs} [$M^{-1}s^{-1}$]
R1	$H^{\bullet} + O_2 \rightarrow HO_2^{\bullet}$	1.27×10^{10}	R31	$e_{aq}^{-} + HO_2^{\bullet} \rightarrow HO_2^{-}$	1.28×10^{10}
R2	$e_{aq}^{-} + O_2 \rightarrow O_2^{\bullet-}$	1.48×10^{10}	R32	$OH^{-} + HO_2^{\bullet} \rightarrow O_2^{\bullet-} + H_2O$	1.27×10^{10}
R3	$^{\bullet}OH + {}^{\bullet}OH \rightarrow H_2O_2$	4.40×10^{9}	R33	$OH^{-} + O(^3P) \rightarrow HO_2^{-}$	4.20×10^{8}
R4	$^{\bullet}OH + e_{aq}^{-} \rightarrow OH^{-}$	2.95×10^{10}	R34	$O_2 + O(^3P) \rightarrow O_3$	4.00×10^{9}
R5	$^{\bullet}OH + OH^{-} \rightarrow O^{\bullet-} + H_2O$	1.27×10^{10}	R35	$O_2 + O^{\bullet-} \rightarrow O_3^{\bullet-}$	3.70×10^{9}
R6	$^{\bullet}OH + O_2^{\bullet-} \rightarrow O_2 + OH^{-}$	1.02×10^{10}	R36	$HO_2^{\bullet} + HO_2^{\bullet} \rightarrow H_2O_2 + O_2$	6.75×10^{5}
R7	$H^{\bullet} + H^{\bullet} \rightarrow H_2$	5.03×10^{9}	R37	$HO_2^{\bullet} + O_2^{\bullet-} \rightarrow HO_2^{-} + O_2$	7.48×10^{7}
R8	$H^{\bullet} + e_{aq}^{-} \rightarrow H_2 + OH^{-}$	2.65×10^{10}	R38	$HO_2^{-} + O(^3P) \rightarrow O_2^{\bullet-} + {}^{\bullet}OH$	5.30×10^{9}
R9	$H^{\bullet} + O(^3P) \rightarrow OH^{\bullet}$	2.00×10^{10}	R39	$e_{aq}^{-} + e_{aq}^{-} \rightarrow H_2 + 2OH^{-}$	6.36×10^{9}
R10	$H^{\bullet} + O^{\bullet-} \rightarrow OH^{-}$	2.00×10^{10}	R40	$H_3O^{+} + OH^{-} \rightarrow H_2O$	1.13×10^{11}
R11	$^{\bullet}OH + O(^3P) \rightarrow HO_2^{\bullet}$	2.00×10^{10}	R41	$H_3O^{+} + O_3^{\bullet-} \rightarrow {}^{\bullet}OH + O_2$	9.00×10^{10}
R12	$HO_2^{\bullet} + O(^3P) \rightarrow O_2 + {}^{\bullet}OH$	2.00×10^{10}	R42	$e_{aq}^{-} + H_3O^{+} \rightarrow H^{\bullet}$	2.11×10^{10}
R13	$O(^3P) + O(^3P) \rightarrow O_2$	2.20×10^{10}	R43	$e_{aq}^{-} + O_2^{\bullet-} \rightarrow H_2O_2 + 2OH^{-}$	1.28×10^{10}
R14	$H^{\bullet} + {}^{\bullet}OH \rightarrow H_2O$	1.44×10^{10}	R44	$e_{aq}^{-} + HO_2^{-} \rightarrow O^{\bullet-} + OH^{-}$	3.51×10^{9}
R15	$H^{\bullet} + H_2O_2 \rightarrow H_2O + {}^{\bullet}OH$	5.18×10^{7}	R45	$e_{aq}^{-} + O^{\bullet-} \rightarrow 2OH^{-}$	2.31×10^{10}
R16	$H^{\bullet} + OH^{-} \rightarrow H_2O + e_{aq}^{-}$	2.51×10^{7}	R46	$H_3O^{+} + O_2^{\bullet-} \rightarrow HO_2^{\bullet}$	4.78×10^{10}
R17	$H^{\bullet} + HO_2^{\bullet} \rightarrow H_2O_2$	1.00×10^{10}	R47	$H_3O^{+} + HO_2^{-} \rightarrow H_2O_2$	4.78×10^{10}
R18	$H^{\bullet} + O_2^{\bullet-} \rightarrow HO_2^{-}$	1.00×10^{10}	R48	$H_3O^{+} + O^{\bullet-} \rightarrow {}^{\bullet}OH$	4.78×10^{10}
R19	$^{\bullet}OH + H_2O_2 \rightarrow HO_2^{\bullet} + H_2O$	2.88×10^{7}	R49	$O_2^{\bullet-} + O^{\bullet-} \rightarrow O_2 + 2OH^{-}$	6.00×10^{8}
R20	$^{\bullet}OH + H_2 \rightarrow H^{\bullet} + H_2O$	4.17×10^{7}	R50	$HO_2^{-} + O^{\bullet-} \rightarrow O_2^{\bullet-} + OH^{-}$	3.50×10^{8}
R21	$^{\bullet}OH + HO_2^{\bullet} \rightarrow O_2 + H_2O$	9.79×10^{9}	R51	$O^{\bullet-} + O^{\bullet-} \rightarrow H_2O_2 + 2OH^{-}$	9.00×10^{8}
R22	$^{\bullet}OH + HO_2^{-} \rightarrow HO_2^{\bullet} + OH^{-}$	8.32×10^{9}	R52	$O^{\bullet-} + O_3^{\bullet-} \rightarrow 2O_2^{\bullet-}$	7.00×10^{8}
R23	$^{\bullet}OH + O^{\bullet-} \rightarrow HO_2^{-}$	7.61×10^{9}	R53	$HO_2^{\bullet} \rightarrow H_3O^{+} + O_2^{\bullet-}$	7.15×10^{5}*
R24	$^{\bullet}OH + O_3^{\bullet-} \rightarrow HO_2^{\bullet} + O_2^{\bullet-}$	8.50×10^{9}	R54	$O_3^{\bullet-} \rightarrow O^{\bullet-} + O_2$	2.66×10^{3}*
R25	$H_2O_2 + e_{aq}^{-} \rightarrow OH^{-} + {}^{\bullet}OH$	1.41×10^{10}	R55	$H^{\bullet} \rightarrow e_{aq}^{-} + H_3O^{+}$	5.94*
R26	$H_2O_2 + OH^{-} \rightarrow HO_2^{-} + H_2O$	1.27×10^{10}	R56	$e_{aq}^{-} + H_2O \rightarrow H^{\bullet} + OH^{-}$	15.8
R27	$H_2O_2 + O(^3P) \rightarrow HO_2^{\bullet} + {}^{\bullet}OH$	1.27×10^{6}	R57	$O_2^{\bullet-} + H_2O \rightarrow HO_2^{\bullet} + OH^{-}$	0.15
R28	$H_2O_2 + O^{\bullet-} \rightarrow HO_2^{\bullet} + OH^{-}$	5.55×10^{8}	R58	$HO_2^{-} + H_2O \rightarrow H_2O_2 + OH^{-}$	1.36×10^{6}
R29	$H_2 + O(^3P) \rightarrow H^{\bullet} + {}^{\bullet}OH$	4.80×10^{3}	R59	$O(^3P) + H_2O \rightarrow 2\,{}^{\bullet}OH$	1.90×10^{3}
R30	$H_2 + O^{\bullet-} \rightarrow H^{\bullet} + OH^{-}$	1.21×10^{8}	R60	$O^{\bullet-} + H_2O \rightarrow {}^{\bullet}OH + OH^{-}$	1.36×10^{6}

S2. Reflective boundary conditions for molecular motion

Due to the long duration (1,000 s) of a water radiolysis simulation in UHDR irradiation, the molecular species were expected to reach the boundary of the 2 × 2 × 2 μm^3 volume repeatedly. Thus, we applied a reflective condition to the box boundaries. When a molecular species reached a box boundary, its motion changed in the manner described in previous studies[3,4]. We considered that molecular species confined in the 1-dimensional space describing $R_{\min} \leq x \leq R_{\max}$ and $L = R_{\max} - R_{\min}$. A new position x_{new} is calculated as follows:

$$x_{\text{new}} = R_{\min} + h \cdot L + (1 - 2h) \cdot |(x - R_{\min}) \bmod L|, \quad (1)$$

and

$$h = \text{trunc}\left(\frac{|x - R_{\min}|}{L}\right) \bmod 2, \quad (2)$$

where $\text{trunc}(x)$ is the truncation operator; thus, h can take a value of 0 or 1. The efficacy of the reflective boundary condition implemented in MPEXS2.1-DNA was verified by comparing the time evolution of the number of hydroxyl radicals simulated with the analytical solution, where the second-order reaction of $^{\bullet}$OH + $^{\bullet}$OH → H_2O_2 (R3 in Table S2) was considered. The differential rate law is proportional to the square of the concentration of hydroxyl radicals:

$$\frac{1}{2}\frac{d[\,^{\bullet}\text{OH}]}{dt} = -k[\,^{\bullet}\text{OH}]^2 \;. \quad (3)$$

This can be used to derive the number of hydroxyl radicals, $N(t)$, at a given time t:

$$N(t) = \frac{1}{\frac{1}{N_0} + \frac{2kt}{N_A V}}, \quad (4)$$

where V is the target volume, N_0 is the initial number of hydroxyl radicals in V, N_A is the Avogadro's number, and k is the reaction rate constant (4.4 × 10^9 [M^{-1}s^{-1})]; R3 in Table S2). Hydroxyl radicals were homogeneously distributed in the target volume. The initial number of hydroxyl radicals was set to 1,000. To verify chemistry simulations from 1 ms to 1,000 s, two types of target volume with dimensions of 3 × 3 × 3 μm^3 and 100 × 100 × 100 μm^3 were used. As mentioned in Section 2.A of the main document, a fixed time step for molecule transportation was employed in the GFDE-SBS model. We discretized the simulation time using logarithmic time steps (Table S3). To reproduce the analytical solution, we adjusted the number of time steps per order of magnitude and each period. This time step configuration was also used in water radiolysis simulations under UHDR conditions. As can be seen in Fig. S1, the MPEXS2.1-DNA simulation results

with the reflective boundary condition were in good agreement with analytical solutions. Thus, we concluded that this boundary condition works well.

Table S3: Settings of logarithmic time steps for transportation of molecular species utilizing the GFDE-SBS model in MPEXS2.1-DNA under UHDR irradiation.

Period	Number of time steps for order of magnitude	Total number of steps for each period
1 ps – 1 μs	40	240
1 μs – 1 ms	240	720
1 ms – 1 s	1,440	4,320
1 s – 10 s	8,640	8,640
10 s – 100 s	25,920	25,920
100 s – 1000 s	77,760	77,760

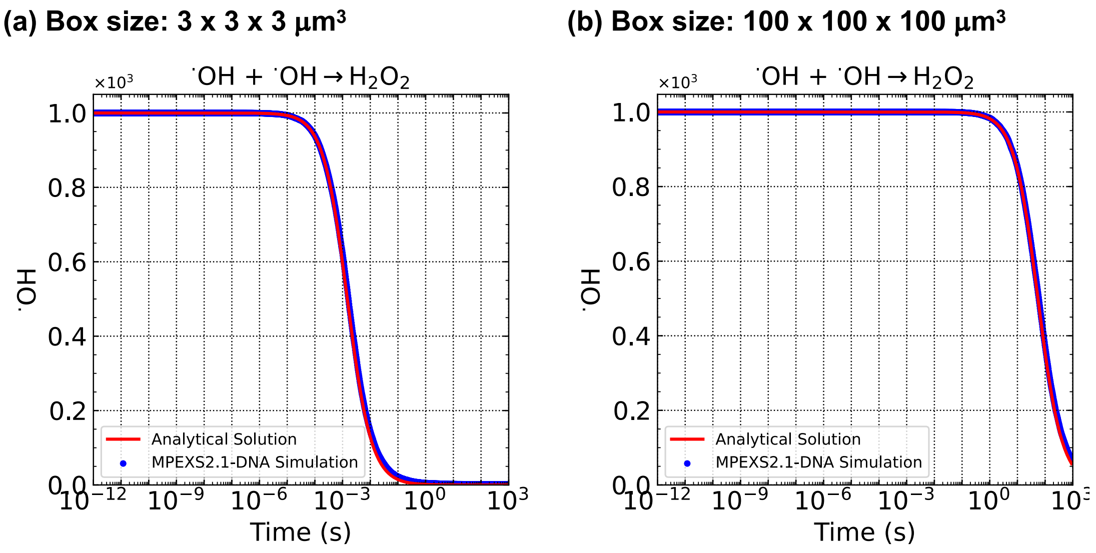

Fig. S1: Comparison between the GFDE-SBS model with the reflective boundary condition (blue filled circles) and the analytical solution (red solid lines) for the chemical reaction of •OH + •OH → H_2O_2 in two target volumes: (a) 3 × 3 × 3 μm^3, and (b) 100 × 100 × 100 μm^3.

S3. Intertrack reactions under UHDR irradiation

This section highlights the characteristics of the reactions of hydroxyl radicals ($^{\bullet}OH$) and hydrogen peroxide (H_2O_2) under UHDR irradiation, as revealed by Monte Carlo simulations.

S3.1 Hydroxyl radicals

We confirmed that $^{\bullet}OH$ was primarily consumed through the four reactions (R3–R6 in Table S2) involving $^{\bullet}OH$, e_{aq}^{-}, OH^{-}, and $O_2^{\bullet-}$. The contribution of each reaction was evaluated by counting the number of $^{\bullet}OH$ that reacted with the species over time under continuous proton irradiation at four different dose rates (0.02, 5, 50, and 500 Gy/s) under the oxygenated condition (pO_2 = 25%) (Fig. S2). Negative values indicate the consumption of $^{\bullet}OH$. Each subplot shows the time evolution of reaction yields at different dose rates, illustrating that the yields of R3 to R6 increase, indicating rapid $^{\bullet}OH$ consumption, with increasing dose rate, and then saturate after several milliseconds. These trends help explain the dose rate dependence of G($^{\bullet}OH$) presented in Figure 4 of the main document.

S3.2 Hydrogen peroxide

Hydrogen peroxide is primarily generated through the recombination of hydroxyl radicals themselves (see R3 in Table S2); thereby, the reactions of R4–R6 are competitive with R3. The yields of R4 and R5 show no differences among dose rate cases (Fig. S2-b and S2-c). In comparison, the yields of R6 become larger, reducing the number of precursors for H_2O_2 production (i.e, $^{\bullet}OH$), at lower dose rates (Fig. S2-d). A previous simulation study [5] also reported that $O_2^{\bullet-}$ persisted in the oxygenated water after pulse irradiation so that hydroxyl radicals were effectively consumed, reducing the yields of hydrogen peroxide. As a result, we observed that the recombination of hydroxyl radicals decreased at lower dose rates (Fig. S2-a). Therefore, calculated G(H_2O_2) increased with increasing dose rates, as shown in Figure 6 in the main document.

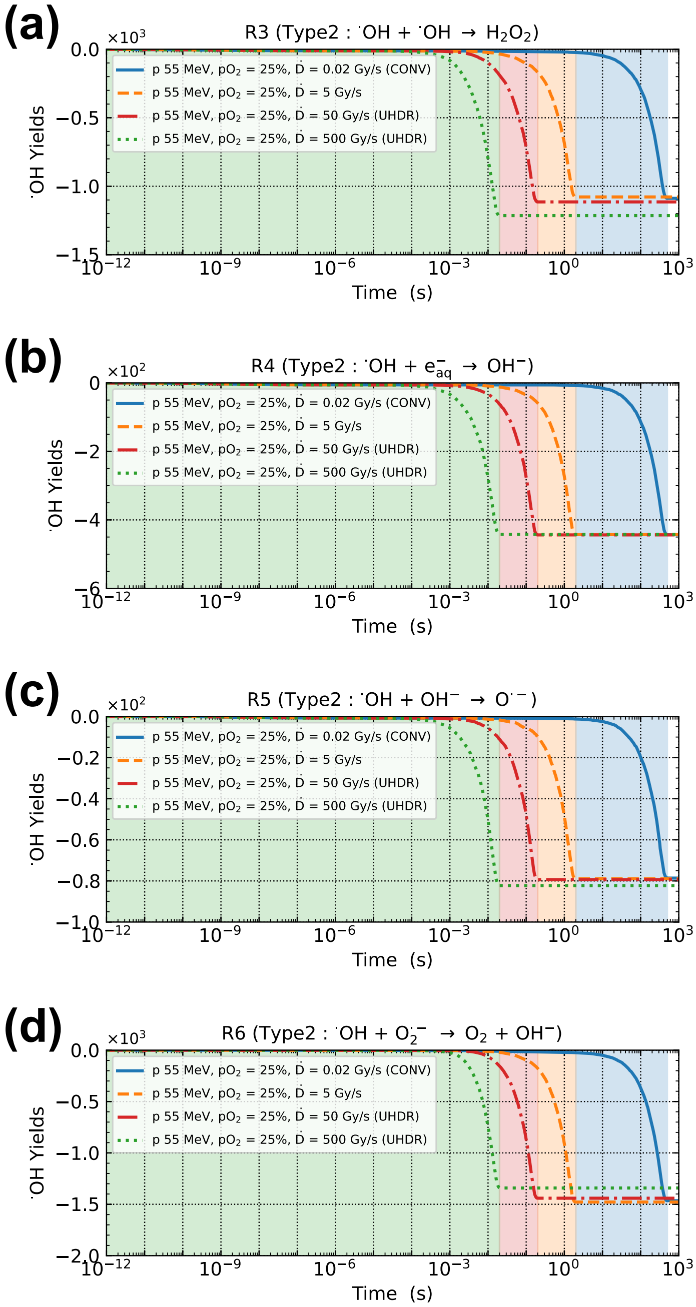

Fig. S2: The time profiles of the occurrence of four reactions (R3–R6) that consume OH radicals under 55 MeV proton irradiation at $\dot{D}$ = 0.02 Gy/s (blue solid line), $\dot{D}$ = 5 Gy/s (orange dashed line), $\dot{D}$ = 50 Gy/s (red dashed-dotted line), and $\dot{D}$ = 500 Gy/s (green dotted line) under the oxygenated condition ($pO_2 = 25\%$). The shaded regions represent the duration of continuous proton irradiation for each dose rate case: blue for 0.02 Gy/s (t_p = 500 s), orange for 5 Gy/s (t_p = 2 s), red for 50 Gy/s (t_p = 200 ms) and green for 500 Gy/s (t_p = 20 ms).

References

1. Okada S, Murakami K, Kusumoto T, Hirano Y, Amako K, Sasaki T. Recent updates of the MPEXS2.1-DNA Monte Carlo code for simulations of water radiolysis under ion irradiation. *Scientific Reports.* 2025;15(1):16534.
2. Frongillo Y, Goulet T, J. Fraser M, Cobut V, P. Patau J, P. Jay-Gerin J. *Monte Carlo simulation of fast electron and proton tracks in liquid water - II. Nonhomogeneous chemistry.* Vol 51, 1998.
3. Karamitros M, Brown J, Lampe N, et al. Implementing the Independent Reaction Time method in Geant4 for radiation chemistry simulations. 2020. doi: 10.48550/arXiv.2006.14225:arXiv:2006.14225. doi:10.48550/arXiv.2006.14225. https://ui.adsabs.harvard.edu/abs/2020arXiv200614225K Accessed June 01, 2020.
4. Tran HN, Chappuis F, Incerti S, Bochud F, Desorgher L. Geant4-DNA Modeling of Water Radiolysis beyond the Microsecond: An On-Lattice Stochastic Approach. *International Journal of Molecular Sciences.* 2021;22(11).
5. Peter W. Radiotherapy Using High-Intensity Pulsed Radiation Beams (FLASH): A Radiation-Chemical Perspective. *Radiation Research.* 2020;194(6):607-617.
